# Effects of exogenous auxin on yield in foxtail millet (*Setaria italica* L.) when applied at the grain-filling stage

**DOI:** 10.3389/fpls.2022.1019152

**Published:** 2023-01-04

**Authors:** Zhiwei Feng, Jing Zhao, Mengen Nie, Fei Qu, Xin Li, Juanling Wang

**Affiliations:** Shanxi Agricultural University, Taiyuan, Shanxi, China

**Keywords:** foxtail millet, NAA, yield, photosynthesis, physiological characteristics

## Abstract

Foxtail millet (*Setaria italica* L.) is of high nutritious value, which is an important crop in arid and semi-arid regions. The objective of this experiment was to explore the effects of the synthetic auxin naphthalene acetic acid (NAA) on the physiological processes of foxtail millet, and to provide a theoretical basis and technical approaches for its efficient use in millet cultivation. Two foxtail millet varieties (‘Jingu 21’ and ‘Zhangzagu 5’) were treated with six concentrations of NAA from 0-144 mg L^-1^ at the grain-filling stage in field experiments. The photosynthetic pigment contents, gas exchange parameters, chlorophyll fluorescence parameters, and grain yield were measured in foxtail millet. The results showed that low concentrations of NAA (18-36 mg L^-1^) increased the contents of photosynthetic pigments, and increased the activities of antioxidant enzymes, the photosynthetic rate, and the activity of photosystem system II (PS II). At higher NAA concentrations, the facilitation effect of the treatments diminished, showing a clear concentration effect. In this study, yield was significantly and positively correlated with PS II effective quantum yield (Y(II)) and the PSII electron transport rate (ETR), and the net photosynthetic rate (Pn) was significantly and positively correlated with chlorophyll content, stomatal conductance (Gs), Y(II), and ETR. These results also indicated that exogenous NAA application promotes the production of ATP and NADPH by increasing the efficiency of electron transfer within the photosystems and also improved photochemical utilization, which facilitates the fixation and reduction of carbon, ultimately leading to an increase in Pn and increasing grain yield in foxtail millet.

## 1 Introduction

Foxtail millet (*Setaria italica* L.) is a nutrient-rich cereal grain that is traditionally grown in arid and semi-arid areas, as it is well adapted to dry climates. In recent years, with the development and utilization of foxtail millet nutrients, the demand for foxtail millet has increased sharply. However, in the actual production process, grain yields have decreased due to irregular planting techniques, continuous cropping obstacles, and pest infestation. Increasing crop yields, improving crop quality, and enhancing crop resistance through chemical control technologies are some of the key agronomic measures in modern agricultural planting technology systems (Wang, 2019; Zhang et al., 2021; Cheng et al., 2020). Plant growth regulators are widely used in crop production to improve plant architecture, increase the photosynthetic rate, enhance root function, improve resistance to stress, increase yield, and improve quality (Zhao & Wang, 2012; Huang et al., 2021; Qamar et al., 2022; Dhaka et al., 2022).

Plant growth regulators, which are important biotic factors, are known to play vital roles not only in plant development, but also in inducing plant tolerance to various environmental stresses (Faisal et al., 2020; Faisal and Muhammad, 2021). Naphthalene acetic acid (NAA) is a synthetic auxin plant growth regulator, which can increase the photosynthetic rate, increase chlorophyll synthesis, promote plant growth, and enhance resistance to stress (Hu et al., 2022). Studies have shown that foliar spraying of NAA can regulate the morphogenesis of eggplant leaves by increasing the cell volume and leaf area of palisade tissue and spongy tissue, and improve the biomass and fruit yield per plant (Rohach, 2017). In yam, NAA treatment promotes the conversion of starch to reducing sugars, thereby accelerating the accumulation of photosynthetic assimilates (Wen et al., 2016). Under tissue culture conditions, NAA treatment was found to increase the activity of PSII in leaves of the Brazilian bromeliad *Aechmea blanchetiana* and promote the growth of test tube seedlings (Martins et al., 2018).

Foxtail millet is similar to crops such as sorghum, maize, and rice in that it is a grass in the botanical family Poaceae. Millet has the potential to be ultra-high yielding, but one reason behind the delayed yield breakthrough is the low efficiency of light energy use. Maximizing the utilization of light energy is the main way to improve crop yield, because 90% of crop dry matter is derived from the products of photosynthesis (Makino, 2011). The efficiency of light energy utilization in foxtail millet populations, especially changes in the overall photosynthetic rate and duration of the upper and middle leaves, has an important influence on the improvement of foxtail millet yields (Fang et al., 2016; Li P. et al., 2019; Liu et al., 2020). There are few studies on the role of NAA in the regulation of photosynthesis and its mechanisms. In this study, the photosynthetic gas exchange parameters and chlorophyll fluorescence parameters of foxtail millet leaves were measured and analyzed following foliar application of NAA at the grain filling stage to investigate the physiological mechanism by which NAA acts to increase yield from the perspective of photosynthesis parameters.

## 2 Materials and methods

### 2.1 Experimental design

Naphthalene acetic acid (20%, powder) was provided by Sichuan Province Guoguang Agrochemical Co., Ltd. The millet varieties used in this experiment were ‘Zhangzagu 5’ and ‘Jingu 21’. Seeds of ‘Zhangzagu 5’ was supplied by the Zhangjiakou Academy of Agricultural Sciences of Hebei Province, China, and ‘Jingu 21’ was provided by the Shanxi Academy of Agricultural Sciences, China.

The field experiments were conducted at the farm of Shanxi Agricultural University, China, in 2012 and 2013. The study site has a temperate continental climate. The experiment used a split-plot design, with millet varieties as the main plot, and two levels of ‘Zhangzagu 5’ and ‘Jingu 21’. The concentration of NAA was the sub-region, and the six concentrations were 0 (CK), 9 mg L^-1^ (C9), 18 mg L^-1^ (C18), 36 mg L^-1^ (C36), 72 mg L^-1^ (C72), and 144 mg L^-1^ (C144). There was a total of 2 × 6 = 12 treatments, repeated three times, and the plot areas were 3 × 6 = 18 m^2^. Foliar application of NAA solution was started at the beginning of the grain filling period, on 25 and 26 August 2012 at around 16:00 hours; the samples were taken on 5 September and the crop was harvested on 5 October. On 28 and 29 August 2013, a foliar spray of NAA solution was applied at around 16:00 hours, samples were taken on 8 September from the first two leaves and the crop was harvested on 8 October. The effect of foliar application of NAA on the physiological characteristics and yield formation of foxtail millet during the filling period was studied.

### 2.2 Measurements

#### 2.2.1 Photosynthetic pigment contents

The chlorophyll a (Chl a), chlorophyll b (Chl b), total chlorophyll, and carotenoid contents were determined following extraction in 96% ethanol. The penultimate leaf was cut into small pieces, and samples (0.1 g) were placed in 15 ml test tubes containing 10 ml of 96% ethyl alcohol, covered with a rubber stopper, and kept in the dark for at least 24 h until the leaves had turned white. During this period, the tubes were shaken 3 to 4 times. The Chl a, Chl b, and carotenoid concentrations were determined by measuring the absorbances at 470, 649, and 665 nm, respectively using a UV 2400 UV-visible spectrophotometer (Sunny Heng Ping Instrument, LLC. Shanghai, China).

#### 2.2.2 Physiological characteristics

The contents of malondialdehyde (MDA) and soluble protein and the activities of superoxide dismutase (SOD) and peroxidase (POD) were determined by the thiobarbituric acid (TBA) test, the Coomassie brilliant blue method, the nitro blue tetrazolium (NTB) method, and the guaiacol method, respectively (Liu et al., 2017). The activity of nitrate reductase (NR) was determined as follows: freshly picked leaves were weighed in two portions of 0.5 g each and 5 mL phosphate buffer and 5 mL potassium nitrate were added to one sample, and 5 mL phosphate buffer and 5 mL distilled water were added to the other. The samples were evacuated using a vacuum pump for 10 min and then placed at a temperature of 30°C for 30 min. A 2 mL sample of each solution was withdrawn, 4 mL of sulphonamide reagent was added to each, mixed well, and then 4 mL of α-naphthylamine reagent was added. The solutions were shaken and placed in an incubator at 30°C for 30 min. The absorbances were measured at 520 nm in a spectrophotometer and the nitrite ion (NO^2-^) concentration was then calculated.

#### 2.2.3 Photosynthetic gas exchange

The net photosynthetic rate (Pn), transpiration rate (Tr), and stomatal conductance (Gs) were measured with a CI-340 portable photosynthesis system (CID Bio-Science, Inc., USA) between 10:00 and 11:00 hours. The photosynthetically active radiation (PAR) at the leaf surface was ~1,000 ± 50 μmol m^−2^ s^−1^, the temperature of the leaf chamber was 28 ± 2°C, and the ambient CO_2_ concentration was 400 ± 50 μmol mol^−1^.

#### 2.2.4 Chlorophyll fluorescence

The photochemical efficiency (Fv/Fm), quantum yield of regulated energy dissipation in PSII (NPQ), photochemical quenching coefficient (qP), PS II effective quantum yield (Y(II), and PSII electron transport rate (ETR) were measured using a miniaturized pulse-amplitude modulated fluorescence analyzer (Mini-PAM, Walz, Effeltrich, Germany) from 17:00 to 22:00 hours. The applied actinic light had a photosynthetic photon flux density (PPFD) of 500 μmol (photon) m^−2^ s^−1^. The high light flash used to measure saturated fluorescence had a PPFD of 4,000 μmol (photon) m^−2^ s^−1^ and a duration of 0.8 s.

#### 2.2.5 Yield and yield components

The grain yield and yield components ear length, ear diameter, ear grain weight were measured after harvesting using a ruler, a Vernier caliper, and an analytical balance (Mettler-Toledo, LLC. Shanghai, China), respectively.

### 2.3 Statistical analysis

Statistical data were analyzed using Microsoft Office Excel 2010 and SPSS 22.0. Duncan’s test was used to determine the significant differences among the treatments at the same time and variety at a significance level of *p*<0.05.

### 2.4 Transcriptome analysis

The original transcriptome sequencing data were obtained from Tang et al., 2022. The BioProject accession number of the data was PRJEB44706. Quality control of the downloaded transcriptome raw data was performed with FASTP V0.20.1 (Chen et al., 2018). The RNAseq data were analysed as described previously (Zheng et al., 2021).

## 3 Results

### 3.1 Effects of NAA on the contents of photosynthetic pigments in leaves of foxtail millet

Total chlorophyll (total Chl), Chl a, Chl b, and carotenoid (Car) contents in leaves of foxtail millet showed a common trend in that they increased after decreased as the NAA concentration increased in 2012 and 2013 (**Table 1**). In 2013, the photosynthetic pigment contents in ‘Zhangzagu 5’ were higher than in ‘Jingu 21’. Although the changes in chlorophyll and carotenoid contents showed similar trends in both ‘Zhangzagu 5’ and ‘Jingu 21’, the relative degrees of increase and decrease differed between the two varieties. The total Chl, Chl a, and Chl b contents in ‘Zhangzagu 5’ were higher by 18.3, 6.7, and 14%, respectively, in the C18 treatment compared to the CK. The highest carotenoid content was in the C36 treatment, and the total Chl and carotenoid contents in ‘Jingu 21’ were significantly higher by 15.6 and 39.5% compared to the CK.

**Table 1:** Effects of naphthalene acetic acid on the photosynthetic pigment contents in leaves of foxtail millet (mg/L FM).

Year	Variety	Treatment	Chl a	Chl b	Car	Total chl
2012	Zhangzagu 5	C0	3.86 ± 0.09b	1.17 ± 0.07a	0.64 ± 0.35c	5.15 ± 0.11bc
		C9	3.98 ± 0.11b	1.21 ± 0.06a	0.63 ± 0.37c	5.32 ± 0.12b
		C18	4.29 ± 0.19a	1.27 ± 0.09a	0.81 ± 0.38ab	5.79 ± 0.20a
		C36	4.42 ± 0.13a	1.3 ± 0.09a	0.89 ± 0.40a	5.68 ± 0.03a
		C72	4.14 ± 0.14ab	1.25 ± 0.07a	0.74 ± 0.38bc	5.22 ± 0.15bc
		C144	3.97 ± 0.11b	1.13 ± 0.15a	0.66 ± 0.31c	5.06 ± 0.03c
	Jingu 21	C0	2.28 ± 0.17c	0.6 ± 0.07b	0.51 ± 0.2d	2.88 ± 0.12d
		C9	2.33 ± 0.18c	0.67 ± 0.01ab	0.53 ± 0.23cd	3.00 ± 0.20cd
		C18	2.62 ± 0.11ab	0.7 ± 0.01a	0.7 ± 0.28ab	3.32 ± 0.12ab
		C36	2.81 ± 0.05a	0.73 ± 0.01a	0.82 ± 0.32a	3.54 ± 0.05a
		C72	2.51 ± 0.11bc	0.69 ± 0.02a	0.66 ± 0.27bc	3.20 ± 0.11bc
		C144	2.31 ± 0.08c	0.7 ± 0.01a	0.54 ± 0.24cd	3.01 ± 0.09cd
2013	Zhangzagu 5	C0	3.44 ± 0.08c	1.00 ± 0.10a	0.48 ± 0.29a	4.43 ± 0.22cd
		C9	3.62 ± 0.12a	1.06 ± 0.07a	0.55 ± 0.32a	4.68 ± 0.07abc
		C18	3.67 ± 0.02a	1.14 ± 0.11a	0.58 ± 0.33a	4.81 ± 0.16a
		C36	3.64 ± 0.06a	1.13 ± 0.07a	0.61 ± 0.34a	4.77 ± 0.01ab
		C72	3.46 ± 0.11b	1.05 ± 0.11a	0.55 ± 0.3a	4.50 ± 0.00bcd
		C144	3.41 ± 0.12b	0.97 ± 0.05a	0.47 ± 0.29a	4.38 ± 0.20d
	Jingu 21	C0	1.95 ± 0.1b	0.49 ± 0.04c	0.38 ± 0.16c	2.44 ± 0.17b
		C9	2.11 ± 0.11ab	0.53 ± 0.02bc	0.44 ± 0.18b	2.63 ± 0.16ab
		C18	2.24 ± 0.18a	0.61 ± 0.04ab	0.51 ± 0.21ab	2.84 ± 0.28a
		C36	2.19 ± 0.13ab	0.63 ± 0.02a	0.53 ± 0.22a	2.81 ± 0.18ab
		C72	2.09 ± 0.07ab	0.51 ± 0.05c	0.42 ± 0.16b	2.59 ± 0.15ab
		C144	1.99 ± 0.14ab	0.48 ± 0.06c	0.43 ± 0.16b	2.47 ± 0.24ab

Numbers followed by different letters indicate significant differences (P<0.05). The same below.

Chlorophyll a, Chl a; Chlorophyll b, Chl b; Total chlorophyll, Total chl; Carotenoid, Car.

### 3.2 Effects of NAA on physiological characteristics in leaves of foxtail millet

**Figure 1:**
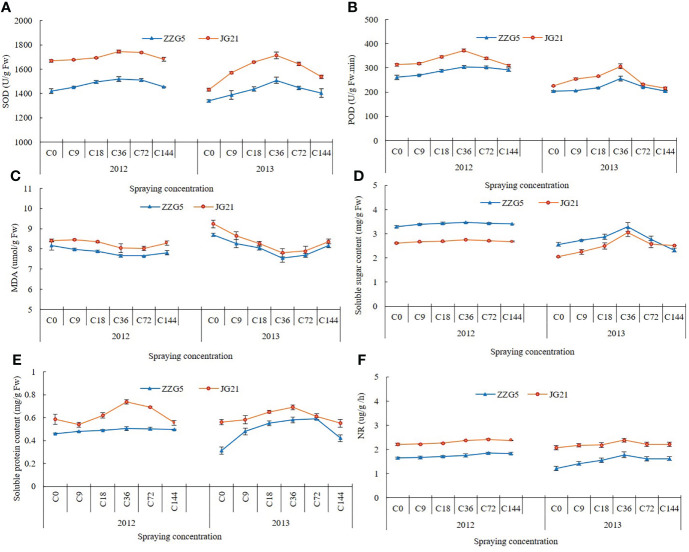
Effects of naphthalene acetic acid on physiological characteristics in leaves of foxtail millet. **(A)** the activity of SOD, **(B)** the activity of POD, **(C)** MDA content, **(D)** soluble sugar content, **(E)** soluble protein content, **(F)** the activity of nitrate reductase.

The SOD activity in both millet varieties showed a trend in which it increased initially and then decreased with increasing concentrations of exogenous NAA, with a peak at 36 mg L^-1^ (**Figure 1A**). The differences between 36 mg L^-1^ and the 0, 9, 18, and 144 mg L^-1^ treatments was significant in 2012 and between 36 mg L^-1^ and the other treatments in 2013. At higher NAA concentrations, the POD activity in both varieties increased and then decreased in both years, peaking at 36 mg L^-1^ (**Figure 1B**). The differences between ‘Zhangzagu 5’ at 36 mg L^-1^ and the 0, 9, and 18 mg L^-1^ treatments in 2012 was significant, while the differences in ‘Jingu 21’ between the 36 mg L^-1^ treatment and the other treatments and between the two varieties at 36 mg L^-1^ and the other treatments in 2013 was significant. The MDA content in both varieties tended to decrease and then increase as the concentration of exogenously applied NAA increased, with the lowest concentration at 36 mg L^-1^ (**Figure 1C**). The differences between 36 mg L^-1^ and the 0 and 9 mg L^-1^ treatments was significant in 2012 as were the differences between 36 mg L^-1^ and 0, 9, 18, and 144 mg L^-1^ in 2013. The soluble sugar content of both varieties tended to increase and then decrease as the concentration of NAA increased, peaking at 36 mg L^-1^ (**Figure 1D**). The difference between 36 mg L^-1^ and the other treatments was significant in 2013, but not in 2012. The results of the two-year trial showed that exogenous application of 36 mg L^-1^ NAA increased in soluble sugar content in foxtail millet. The soluble protein content of the two varieties showed a trend of increasing and then decreasing with the increase of NAA concentration, reaching a peak at 36 mg L^-1^ (**Figure 1E**). In 2012, the difference between the concentration of 36 mg L^-1^ and the other treatments was significant for ‘Jingu 21’, while there was no significant difference between all treatments for ‘Zhangzagu 5’. The nitrate reductase activity of the two varieties showed a trend of increasing and then decreasing with the increase of NAA concentration (**Figure 1F**). In 2012, the nitrate reductase activity did not change significantly with the increase of external application concentration and reached the maximum value at 72 mg L^-1^, but the difference with other treatments was not significant.

### 3.3 Effects of NAA on gas exchange parameters in leaves of foxtail millet

The Pn, Tr and Gs of ‘Zhangzagu 5’ and ‘Jingu 21’ all tended to increase and then decrease with increasing NAA concentration (**Table 2**). As the treatment concentration increased, the facilitation effect diminished, showing a clear concentration effect. Pn, Tr and Gs values of ‘Zhangzagu 5’ and ‘Jingu 21’ were greatest at 18 mg L^-1^ with significant increases of 14.5, 10.9, 9.5 and 10.7, 6.7, 10.0% respectively over the control.

**Table 2:** Effects of naphthalene acetic acid on gas exchange parameters in leaves of foxtail millet.

Year	Variety	Treatment	Pn (μmol CO_2_ m^-2^ s^-1^)	Tr (mmol H_2_O m^-2^ s^-1^)	Gs (mmol H_2_O m^-2^ s^-1^)
2012	Zhangzagu 5	C0	16.12 ± 1.46c	2.20 ± 0.34a	85.54 ± 1.09c
		C9	16.40 ± 0.76c	2.19 ± 0.08a	85.30 ± 0.54c
		C18	17.80 ± 0.08a	2.20 ± 0.13a	87.35 ± 0.57b
		C36	18.14 ± 0.3a	2.23 ± 0.01a	89.45 ± 0.58a
		C72	17.69 ± 0.35ab	2.22 ± 0.01a	88.16 ± 0.57b
		C144	16.91 ± 0.05bc	2.16 ± 0.02a	87.89 ± 0.57b
	Jingu 21	C0	18.59 ± 0.42d	2.95 ± 0.05bc	104.6 ± 5.69c
		C9	18.25 ± 0.25d	2.92 ± 0.04c	105.76 ± 3.25bc
		C18	20 ± 0.07ab	2.97 ± 0.04b	107.31 ± 0.31abc
		C36	20.34 ± 0.29a	3.12 ± 0.04a	109.91 ± 0.38a
		C72	19.89 ± 0.33b	3.08 ± 0.02a	109.12 ± 1.10ab
		C144	19.11 ± 0.06c	2.96 ± 0.04bc	107.85 ± 0.31abc
2013	Zhangzagu 5	C0	13.30 ± 0.05d	1.75 ± 0.01bc	79.49 ± 0.32b
		C9	14.67 ± 0.06ab	1.85 ± 0.01ab	84.21 ± 0.33b
		C18	15.23 ± 0.20a	1.94 ± 0.02a	87.08 ± 1.12a
		C36	14.12 ± 0.43bc	1.89 ± 0.06a	84.76 ± 2.60b
		C72	13.61 ± 0.29cd	1.78 ± 0.04bc	82.82 ± 1.79b
		C144	13.05 ± 0.68d	1.69 ± 0.09c	83.33 ± 0.5b
	Jingu 21	C0	15.84 ± 0.09c	2.54 ± 0.01c	96.10 ± 0.54c
		C9	16.77 ± 0.17b	2.64 ± 0.03ab	102.34 ± 1.05b
		C18	17.53 ± 0.33a	2.71 ± 0.05a	105.67 ± 2.02a
		C36	17.01 ± 0.26b	2.66 ± 0.04a	101.39 ± 1.56b
		C72	16.08 ± 0.30c	2.57 ± 0.05bc	97.52 ± 1.83c
		C144	15.62 ± 0.11c	2.51 ± 0.02c	95.18 ± 0.65c

Net photosynthetic rate, Pn; Transpiration rate, Tr; Stomatal conductance, Gs.

### 3.4 Effects of NAA on chlorophyll fluorescence in foxtail millet leaves

The maximum photochemical quantum efficiency (Fv/Fm) is used to measure the primary light energy conversion efficiency of photosystem II (PSII) in plant leaves, and reflects the ability of PSII to use light. The Fv/Fm of PSII in the leaves of both ‘Zhangzgu 5’ and ‘Jingu 21’ ranged from 0.75-0.78 in the six NAA treatments in 2012 and 2013, indicating that the plants grew normally and were not under stress (**Table 3**). Y(II) reflects the actual photosynthetic efficiency of the plant leaves. In the C36 treatment, Y(II) was significantly higher by 29.2% in ‘Zhangzagu 5’ compared to the CK, and the difference between NAA treatments was not significant in 2013. Y(II) was highest in ‘Jingu 21’ in the C72 treatment with a significant increase of 8.0% compared to the CK, but it was significantly lower in the C144 treatment. ETR is used to measure the electron transfer efficiency of carbon fixation caused by photochemical reactions, and reflects the speed of apparent electron transfer. In the C18 and C36 treatments, the apparent electron transfer in leaves of ‘Zhangzagu 5’ was faster, and was significantly higher than in the other treatments. In ‘Jingu 21’, the ETR was highest in the C36 treatment, and it showed a significant increase of 10.5% compared with the CK; ETR in the C144 treatment was significantly lower than in the CK. qP reflects the level of photosynthetic activity of the plant and is proportional to the electron transfer activity of the photosystems. The qP in ‘Jingu 21’ in the C9 treatment was the highest, and it was significantly higher than in the CK. NPQ reflects the ability of the plant to dissipate excess light energy in the form of heat, a self-protective capability of the photosystems. The smaller the NPQ, the better the plant’s ability to use light energy. In the C36 treatment, NPQ was lowest in both ‘Zhangzagu 5’ and ‘Jingu 21’, 13.1 and 4.0% lower than the CKs, respectively. Taken together, the photosynthetic capacity of ‘Zhangzagu 5’ and ‘Jingu 21’ leaves in all NAA treatments was the best in the C36 treatment, which had the highest photosynthetic capacity and significantly improved photosynthetic utilization, reducing NPQ and improving light energy utilization under normal light conditions. Pn showed a significant positive correlation with Y(II) and ETR, and a negative correlation with NPQ, demonstrating that NAA enhances the net photosynthetic capacity by promoting electron transport and photochemical utilization in foxtail millet leaves.

**Table 3:** Effects of naphthalene acetic acid on chlorophyll fluorescence in leaves of foxtail millet.

Year	Variety	Treatment	Fv/Fm	Y(II)	qP	NPQ	ETR
2012	Zhangzagu 5	C0	0.7768 ± 0.0183a	0.301 ± 0.007b	0.529 ± 0.012b	1.65 ± 0.04a	138.4 ± 3.26cd
		C9	0.7694 ± 0.0066a	0.319 ± 0.003a	0.558 ± 0.005a	1.53 ± 0.01b	147.81 ± 1.27b
		C18	0.7791 ± 0.0074a	0.323 ± 0.003a	0.555 ± 0.005a	1.53 ± 0.01b	153.19 ± 1.46a
		C36	0.7808 ± 0.0159a	0.33 ± 0.007a	0.554 ± 0.011a	1.42 ± 0.03c	154.91 ± 3.16a
		C72	0.772 ± 0.0026a	0.307 ± 0.005b	0.533 ± 0.009b	1.52 ± 0.02b	141.76 ± 2.31c
		C144	0.7718 ± 0.0003a	0.304 ± 0b	0.544 ± 0ab	1.67 ± 0a	136.67 ± 0.06d
	Jingu 21	C0	0.7831 ± 0.0002a	0.308 ± 0.001c	0.549 ± 0.004c	1.76 ± 0.01a	144.75 ± 1.18c
		C9	0.7851 ± 0.0013a	0.342 ± 0.001ab	0.599 ± 0.001a	1.75 ± 0a	161.68 ± 0.27ab
		C18	0.7899 ± 0.0082a	0.336 ± 0.003b	0.579 ± 0.006b	1.74 ± 0.02ab	159.7 ± 1.65b
		C36	0.7894 ± 0.0049a	0.342 ± 0.002ab	0.591 ± 0.004a	1.71 ± 0.01b	162.83 ± 1.01a
		C72	0.7876 ± 0.0064a	0.343 ± 0.003a	0.6 ± 0.005a	1.78 ± 0.01a	160.54 ± 1.3ab
		C144	0.7834 ± 0.0004a	0.292 ± 0.002d	0.517 ± 0.004d	1.79 ± 0.01a	138.42 ± 1.13d
2013	Zhangzagu 5	C0	0.7544 ± 0.0199a	0.292 ± 0.008b	0.514 ± 0.014a	1.6 ± 0.04a	134.41 ± 3.55c
		C9	0.7613 ± 0.0234a	0.316 ± 0.01a	0.552 ± 0.017a	1.51 ± 0.05b	146.25 ± 4.5b
		C18	0.77 ± 0.0082a	0.319 ± 0.003a	0.549 ± 0.006a	1.52 ± 0.02b	151.4 ± 1.61a
		C36	0.7613 ± 0.0337a	0.322 ± 0.014a	0.54 ± 0.024a	1.39 ± 0.06c	151.04 ± 6.69a
		C72	0.7688 ± 0.0255a	0.301 ± 0.01ab	0.522 ± 0.017a	1.48 ± 0.05c	138.92 ± 4.6bc
		C144	0.7714 ± 0.0315a	0.304 ± 0.012ab	0.544 ± 0.022a	1.67 ± 0.07a	136.6 ± 5.58bc
	Jingu 21	C0	0.7833 ± 0.0109a	0.314 ± 0.004b	0.554 ± 0.008c	1.77 ± 0.02a	146.2 ± 2.03b
		C9	0.7835 ± 0.0053a	0.341 ± 0.002a	0.598 ± 0.004a	1.75 ± 0.01a	161.35 ± 1.09a
		C18	0.7799 ± 0.0082a	0.332 ± 0.004a	0.572 ± 0.006bc	1.71 ± 0.02b	157.68 ± 1.67a
		C36	0.7834 ± 0.0136a	0.339 ± 0.006a	0.587 ± 0.01ab	1.7 ± 0.03b	161.59 ± 2.8a
		C72	0.7798 ± 0.0162a	0.34 ± 0.007a	0.594 ± 0.012a	1.76 ± 0.04a	158.95 ± 3.3a
		C144	0.7839 ± 0.0113a	0.295 ± 0.004c	0.522 ± 0.008d	1.81 ± 0.03a	139.8 ± 2.02c

Photochemical efficiency, Fv/Fm; Quantum yield of regulated energy dissipation in PSII, NPQ; PS II effective quantum yield, Y(II); PSII electron transport rate, ETR; photochemical quenching coefficient, qP.

### 3.5 Effects of NAA on the yield and yield components of foxtail millet

In both 2012 and 2013, the spike length, ear diameter, 1,000-grain weight, ear grain weight, and yield in ‘Zhangzagu 5’ and ‘Jingu 21’ all increased and then decreased with increasing concentrations of NAA (**Table 4**). The highest yields were achieved in the C36 treatment for ‘Zhangzagu 5’ and’ Jingu 21’, which were 5.8 and 7.7% higher, respectively, than the CK in 2012, and 7.9 and 9.0% higher than the CK, respectively, in 2013. The increase in yield was slightly greater in 2013 than in 2012.

**Table 4:** Effects of naphthalene acetic acid on the yield and yield components of foxtail millet.

Year	Variety	Treatment	Ear length (cm)	Ear diameter (mm)	1000-grain Weight (g)	Ear grain weight (g)	Yield (kg/hm ^2^)
2012	Zhangzagu 5	C0	25.51 ± 0.24c	30.25 ± 0.16d	3.1 ± 0.08c	21.71 ± 0.32d	6111.48 ± 46.13c
		C9	26.58 ± 0.28b	31.51 ± 0.25bc	3.21 ± 0.02b	24.33 ± 0.23c	6265.46 ± 48.05bc
		C18	27.39 ± 0.18a	31.63 ± 0.15b	3.26 ± 0.02b	24.92 ± 0.12b	6382.53 ± 61.67ab
		C36	27.65 ± 0.26a	32.42 ± 0.18a	3.42 ± 0.01a	26.04 ± 0.12a	6468.15 ± 41.62a
		C72	26.39 ± 0.22b	31.28 ± 0.26bc	3.25 ± 0.02b	24.34 ± 0.09c	6349.18 ± 39.71ab
		C144	25.56 ± 0.11c	31.11 ± 0.04c	3.22 ± 0.03b	22.14 ± 0.06d	6220.69 ± 121.78bc
	Jingu 21	C0	27.5 ± 0.66c	32.04 ± 0.25c	3.17 ± 0.02c	22.2 ± 0.25c	4470.46 ± 57.67c
		C9	28.23 ± 0.3bc	33.63 ± 0.06b	3.31 ± 0.04b	23.7 ± 0.65b	4676.18 ± 53.46b
		C18	28.96 ± 0.53ab	34.67 ± 0.27a	3.57 ± 0.1a	25.31 ± 0.35a	4721.36 ± 45.51ab
		C36	29.71 ± 0.33a	34.85 ± 0.18a	3.62 ± 0.02a	26.41 ± 0.49a	4816.68 ± 56.03a
		C72	28.88 ± 0.45ab	33.69 ± 0.25b	3.42 ± 0.02b	25.57 ± 0.49a	4702.73 ± 57.93ab
		C144	28.47 ± 0.35bc	32.31 ± 0.08c	3.33 ± 0.03b	23.27 ± 0.91bc	4674.26 ± 34.89b
2013	Zhangzagu 5	C0	25.2 ± 0.19b	29.5 ± 0.33c	2.93 ± 0.06d	20.38 ± 0.16c	5452.54 ± 62d
		C9	25.42 ± 0.22b	30.1 ± 0.12bc	3.13 ± 0.09bc	22.94 ± 0.06b	5605.12 ± 76.14c
		C18	26.23 ± 0.49a	30.77 ± 0.27ab	3.27 ± 0.07ab	23.6 ± 0.17b	5767.45 ± 40.57ab
		C36	26.81 ± 0.22a	31.17 ± 0.43a	3.39 ± 0.01a	24.67 ± 0.16a	5882.77 ± 65.32a
		C72	25.35 ± 0.25b	30.06 ± 0.35bc	3.17 ± 0.06bc	23.08 ± 0.1b	5725.18 ± 29.59bc
		C144	25.09 ± 0.11b	29.64 ± 0.32c	3.09 ± 0.07cd	20.6 ± 0.65c	5600.52 ± 48.17c
	Jingu 21	C0	25.39 ± 0.54c	30.2 ± 0.19c	2.94 ± 0.06d	21.74 ± 0.35c	4200.57 ± 26.61d
		C9	26.94 ± 0.5b	31.67 ± 0.3b	3.08 ± 0.08c	22.8 ± 0.37b	4337.18 ± 43.56c
		C18	27.07 ± 0.55b	32.6 ± 0.37a	3.39 ± 0.02a	23.51 ± 0.5b	4416.36 ± 32.27b
		C36	28.71 ± 0.38a	32.81 ± 0.23a	3.48 ± 0.08a	24.96 ± 0.25a	4576.68 ± 28.82a
		C72	26.8 ± 0.23b	31.53 ± 0.33b	3.25 ± 0.01b	23.41 ± 0.51b	4317.73 ± 22.57c
		C144	26.39 ± 0.4bc	30.39 ± 0.32c	3.08 ± 0.02c	21.58 ± 0.45c	4234.26 ± 35.82d

### 3.6 Correlation between photosynthetic characteristics and grain yield in foxtail millet

As shown in **Table 5**, Pn showed a highly significant positive correlation with chlorophyll content and Tr, and a significant positive correlation with Gs, Y(II), and ETR, further indicating that the enhancement of Pn by NAA is related to the improvement of both stomatal and non-stomatal factors, which in turn was closely related to the enhancement of PSII electron transfer efficiency. The 1,000-grain weight was significantly positively correlated with chlorophyll content, Gs, Y(II), and ETR, and was highly significantly positively correlated with yield.

**Table 5:** Correlation between photosynthetic characteristics and yield in foxtail millet.

	Total chl	Pn	Tr	Gs	Ci	Fv/Fm	Y(II)	qP	NPQ	ETR	1000-grain Weight	Yield
Total chl	1											
Pn	0.91**	1										
Tr	0.99**	0.93**	1									
Gs	0.78*	0.79*	0.75	1								
Ci	-0.92**	-0.90**	-0.91**	-0.73	1							
Fv/Fm	0.01	0.11	-0.01	0.6	0.07	1						
Y(II)	0.89**	0.77*	0.81*	0.89**	-0.84*	0.24	1					
qP	0.56	0.63	0.47	0.82*	-0.66	0.44	0.82*	1				
NPQ	-0.75	-0.5	-0.72	-0.41	0.46	0.16	-0.62	-0.1	1			
ETR	0.98**	0.87*	0.94**	0.87*	-0.90**	0.14	0.96**	0.68	-0.71	1		
1000-grain Weight	0.79*	0.56	0.73	0.81*	-0.59	0.36	0.87*	0.51	-0.77*	0.86*	1	
Yield	0.71	0.47	0.66	0.75	-0.47	0.4	0.77*	0.39	-0.78*	0.78*	0.98**	1

*significant at p<0.05.**significant at p<0.01.

## 4 Discussion

Photosynthetic pigments are important in photosynthesis and are not only involved in the absorption of light energy, but also play key roles in the photoelectric conversion process (Kim et al., 2013). The results of our study showed that total Chl content in foxtail millet leaves was significantly increased by NAA treatment at appropriate concentrations. This may be related to the fact that NAA stimulates the growth of foxtail millet roots and promotes the uptake of minerals such as Fe (Prasad et al., 2014), thus facilitating chlorophyll synthesis. NAA may also be directly involved in chlorophyll anabolism. Growth hormone signaling and its response factor SlARF10 are directly involved in the regulation of leaf chlorophyll metabolism during tomato fruit development, and high levels of expression are beneficial for promoting the synthesis and accumulation of chlorophyll (Yuan et al., 2018). The increase in total Chl levels is beneficial to the improvement of photosynthetic performance in foxtail millet. The results of correlation analysis in this study further showed that the total Chl content in foxtail millet leaves is positively correlated with the net photosynthetic rate in the NAA treatments, indicating that the increase in total Chl content was one of the important factors for improving the net photosynthetic capacity of foxtail millet by NAA treatment. Khan et al. (2018) showed that NAA treatment increased the carotenoid content in maize leaves, which is consistent with the results of the present study. In our study, we found that the carotenoid contents of ‘Zhangzagu 5’ and ‘Jingu 21’ were significantly higher by 39.0 and 60.8%, respectively, in the C36 treatment compared to the control. This may be related to the up-regulation of carotenoid synthesis genes and the down-regulation of catabolism genes induced by the NAA treatment. In addition, ethylene production was induced by NAA treatment, and the increase in the endogenous ethylene level could stimulate carotenoid biosynthesis (Ma et al., 2021).

Soluble sugars are the main components of carbohydrates that can be interconverted and reused. They are not only the immediate products of photosynthesis, but also are the main form of carbohydrate metabolism and temporary storage (Wang et al., 2005). Compared with ‘Zhangzagu 5’, ‘Jingu 21’ had a higher photosynthesis rate and a lower leaf soluble sugar content, indicating that ‘Jingu 21’ leaves have a stronger capacity to transport sucrose, which can facilitate the transport of sugars to the seed bank, thus providing sufficient raw material for starch synthesis in the seeds. In this study, we found that the exogenous application of 36 mg/L NAA promoted the accumulation of soluble sugars in the leaves of foxtail millet without affecting normal leaf photosynthesis, resulting in an increase in yield. Our results show that the soluble protein content in millet leaves was significantly higher than in the control after spraying with a narrow concentration range of NAA, indicating that some concentrations of NAA can promote soluble protein synthesis. This is consistent with the results of Jiang et al. (2015) in soybean. Nitrate reductase is a key rate-limiting enzyme in the nitrogen assimilation process in plants (Kazuhisa et al., 2003), and nitrate reductase activity in functional leaves of cereals can affect the plant’s ability to absorb external nitrogen, which ultimately affects crop yield. We showed that exogenous application of a NAA within a certain concentration range could significantly increase the nitrate reductase activity in the millet varieties ‘Jingu 21’ and ‘Zhangzagu 5’. MDA, which is the end product of membrane lipid peroxidation in plants, is an important indicator of the degree of crop damage from oxidative stress (Kumar et al., 2020). SOD and POD are protective enzyme systems in plants that scavenge reactive oxygen species that are produced as by-products of aerobic metabolism and ultimately protect membrane structures (Awan et al., 2021), thus enabling plants to withstand stress. A previous study showed that treatment of sweet pepper seeds with the hormones IBA+NAA significantly enhanced the soluble sugar, soluble protein, and proline contents of seedlings at low temperature, enhanced the activities of protective enzymes, and reduced the degree of membrane lipid peroxidation, thus improving seedling stress tolerance (Qu et al., 2006). In this study, application of suitable concentrations of NAA enhanced the activities of SOD and POD and reduced the MDA content of foxtail millet leaves, improving plant resistance to environmental stresses.

**Figure 2:**
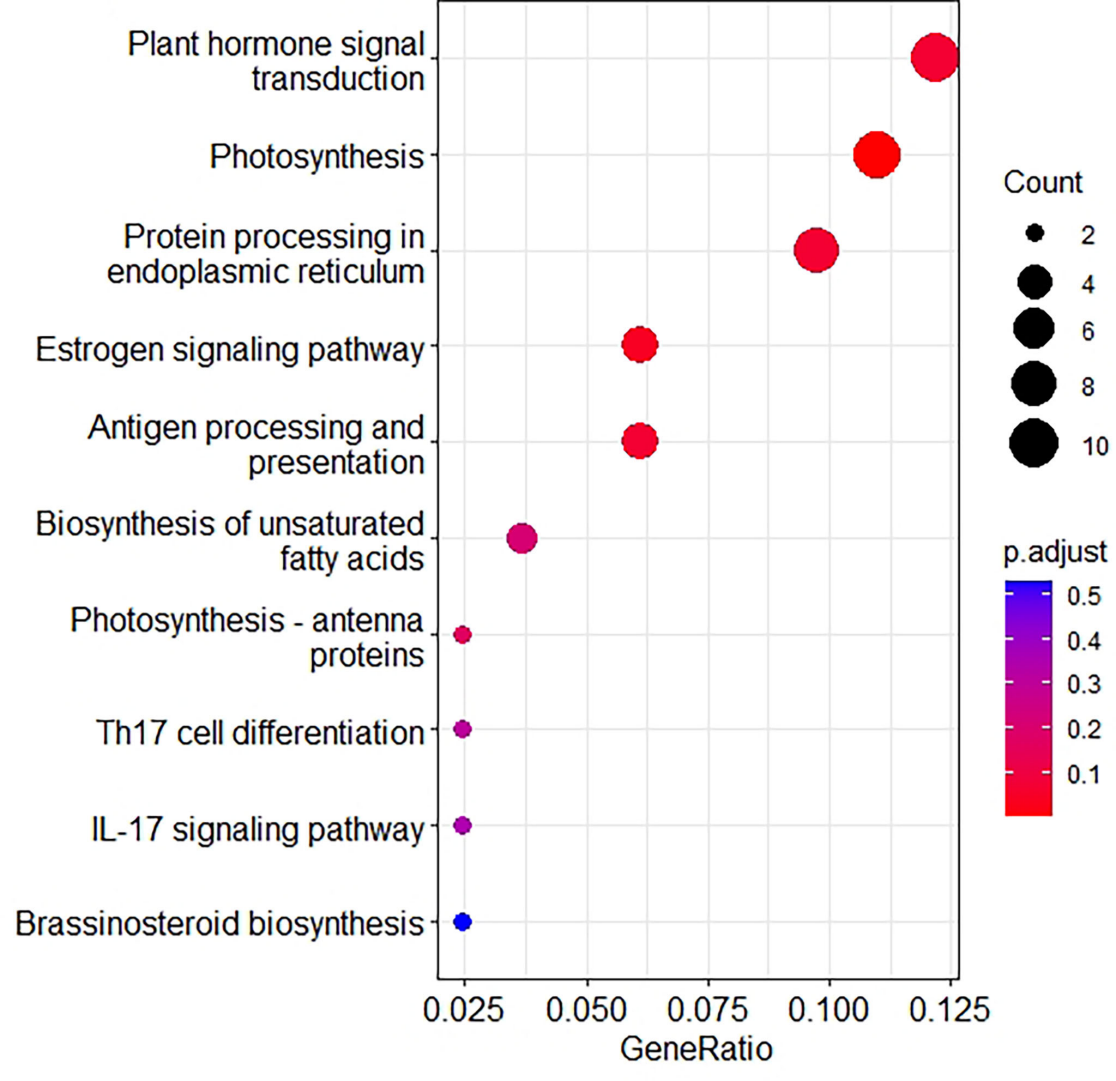
KEGG enrichment analysis of DEGs from loss-of-function AUX1 of foxtail millet.

Previous studies have shown that exogenous application of the auxin indole acetic acid (IAA) can induce stomatal conductance, improve leaf photosynthetic rate, increase sucrose catabolism enzyme activity, and change the distribution of photosynthetic assimilates among different types of carbohydrates in leaves (Cui et al., 2009; Li J. et al., 2019). Furthermore, foliar application of IAA also promotes the export of photosynthetic products from functional leaves and increases the accumulation of soluble sugars in the crop root system. In this study, the increase in Pn and Tr in foxtail millet leaves after treatment with appropriate concentrations of NAA was associated with an increase in Gs. The increase in Gs was accompanied by a decrease in Ci, indicating an increase in the assimilation of CO_2_ by the chloroplasts. The increase in Pn was accompanied by an increase in photosynthetic pigment content and a decrease in Ci, suggesting that photosynthetic pigment content and non-stomatal factors together affect the net photosynthetic rate of cereal leaves, which is similar to the findings of Shi et al. (2021). In addition, from the results showing that Pn and Gs are significantly positively correlated, we found that the enhancement of photosynthetic capacity in millet promoted by NAA treatment was also related to the improvement of stomatal limiting factors. Salehifar et al. (2017) showed that NAA treatment improves the stomatal conductance of plant leaves under drought stress, thereby enhancing the formation of photosynthetic assimilates. Therefore, treatment with suitable concentrations of NAA can improve the photosynthetic performance of cereals by improving both non-stomatal and stomatal factors. Besides, we use the RNA-seq data from a loss-of-function AUX1 (auxin influx carrier) of foxtail millet at heading stage, results showed that auxin was involved in the photosynthesis process (**Figure 2**).

As an internal probe of photosynthesis, chlorophyll fluorescence can respond directly or indirectly to the primary reactions of photosynthesis, electron transfer, and CO_2_ assimilation, and is used to rapidly analyze the absorption, conversion, transfer, and utilization of light energy by the plant photosynthetic system (Matthew et al., 2020). The results of our study showed that suitable concentrations of NAA significantly increased the Fv/Fm, Y(II), ETR, and qP of foxtail millet leaves, while decreasing the NPQ. We showed that NAA can improve the efficiency of capturing primary light energy in the PSII reaction center of foxtail millet. The openness of the PSII reaction center increased, resulting in higher effective light quantum production and an enhanced electron transfer rate, thus promoting the production of ATP and NADPH and facilitating carbon fixation and reduction, ultimately leading to an increase in Pn (Shi et al., 2021). NAA in the C36 treatment was found to reduce the degree of heat dissipation caused by excess excitation energy, which is a reflection of the mitigation of photoinhibition in foxtail millet leaves. This may be due to the fact that NAA treatment promotes growth factor signaling in foxtail millet, resulting in the up-regulation of photosynthesis-related genes, including Chl a/b-binding protein gene expression. The elevated contents of chlorophyll, carotenoids, and other pigments in foxtail millet leaves regulates the pigment signaling process (Guo et al., 2016), protects the structures of reactive enzymes and pigment synthases in the photosynthetic system, promotes photosynthetic electron activity, accelerates the carbon reduction process of photosynthesis (Liang et al., 2017), and improves the photosynthetic performance of foxtail millet leaves.

Yield is the most important indicator of the effect of a cultivation practice or technical tool, and any improvement in yield components will contribute to an increase in overall yield (Xu et al., 2017). Our study showed that treatment with NAA at the appropriate concentration increased spike length, spike thickness, 1,000-grain weight, and spike weight in both foxtail millet varieties and improved grain yield significantly. Plant growth regulators increase yield by expanding sources, increasing pools, and coordinating the balance of carbon and nitrogen metabolism (Luo et al., 2021). In this study, yield was significantly and positively correlated with Y(II) and ETR, and Pn was significantly and positively correlated with chlorophyll content, Gs, Y(II), and ETR. Our results show that exogenous NAA promotes the production of ATP and NADPH by increasing the efficiency of electron transfer within the photosystem and the improvement of photochemical utilization, which facilitates the fixation and reduction of carbon, ultimately leading to an increase in Pn and achieving the effect of increasing dry matter accumulation.

## 5 Conclusions

In conclusion, low concentrations of NAA (18-36 mg L^-1^) applied at the grain-filling stage increased the contents of photosynthetic pigments, the activities of antioxidant enzymes the, photosynthetic rate, and the photosynthetic system II activity. As the NAA concentration in the treatments increased, the facilitation effect diminished, showing a clear concentration effect. Grain yield was significantly and positively correlated with Y(II) and ETR, and Pn was significantly and positively correlated with chlorophyll content, Gs, Y(II), and ETR. Exogenous NAA promotes the production of ATP and NADPH by increasing the efficiency of electron transfer within the photosystem and the improvement of photochemical utilization, which facilitates the fixation and reduction of carbon, ultimately leading to an increase in Pn and an increased yield in foxtail millet.

## Data availability statement

The raw data supporting the conclusions of this article will be made available by the authors, without undue reservation.

## Author contributions

FQ, ZF, and XL designed the experiment. ZF, JZ, and MN collected the samples, analyzed the samples, ZF, JZ, and XL drafted and revised the manuscript, JW was involved in reviewing and editing the revised manuscript and supervision. All authors have read and approved the final manuscript.

## References

[B1] Awan S. A. Khan I. Rizwan M. Zhang X. Brestic M. Khan A.. (2021). Exogenous abscisic acid and jasmonic acid restrain PEG-induced drought by improving the growth and antioxidative enzyme activities in pearl millet. Physiol. Plan 22, 809–819. doi: 10.1111/ppl.13247 33094486

[B2] Cheng F. Y. Wang H. Han Y. Q. Han Y. H. (2020). Research advances on downy mildew of foxtail millet in China. J. Shanxi Agric. Sci. 48 (2), 263–267.

[B3] Chen S. F. Zhou Y. Q. Chen Y. R. Gu J. (2018). Fastp: an ultra-fast all-in-one FASTQ preprocessor. Bioinformatics 34, i884–i890. doi: 10.1093/bioinformatics/bty560 30423086 PMC6129281

[B4] Cui N. Li T. L. Zhao J. Y. Lin F. Bai L. P. (2009). Effects of exogenous auxin on related enzymes activities of sucrose metabolism and gene expression in tomato fruits. Acta Agriculturae Boreali-Sinica 24, 3, 99–101.

[B5] Dhaka A. K. Kamboj N. K. Kumar S.. (2022). Efficacy of plant growth regulators on productivity, quality and profitability of berseem (*Trifolium alexandrium*). Indian J. Agron. 67 (2), 186–191.

[B6] Faisal Z. Adnan Y. Patrick M. F. Antonio F. (2020). Comparison of soaking corms with moringa leaf extract alone or in combination with synthetic plant growth regulators on the growth, physiology and vase life of sword lily. Plants. 9 (11), 1590. doi: 10.3390/plants9111590 33212881 PMC7698385

[B7] Faisal Z. Muhammad A. (2021). Bioregulators: unlocking their potential role in regulation of the plant oxidative defense system. Plant Mol. Biol. 105, 11–41. doi: 10.1007/s11103-020-01077-w 32990920

[B8] Fang X. M. Dong K. J. Wang X. Q. Liu T. He J. Ren R.. (2016). A high density genetic map and QTL for agronomic and yield traits in foxtail millet [*Setaria italica* (L.) p. Beauv]. BMC Genomics 17, 336. doi: 10.1186/s12864-016-2628-z 27146360 PMC4857278

[B9] Gao Z. P. Guo P. Y. Yuan X. Y. Dong S. Q. Liu Y. Gao H.. (2015). Effects of tribenuron-methyl and monosulfuron application on photosynthetic characteristics and yield of zhangza gu 10. J. China Agric. Univ. 20, 36–45.

[B10] Guo Z. X. Wang F. Xiang X. Ahammed G. J. Wang M. Onac E.. (2016). Systemic induction of photosynthesis *via* illumination of the shoot apex is mediated sequentially by phytochrome b, auxin and hydrogen peroxide in tomato. Plant Physiol. 172 (2), 1259–1272. doi: 10.1104/pp.16.01202 27550998 PMC5047115

[B11] Huang G. M. Liu Y. R. Guo Y. L. Peng C. X. (2021). A novel plant growth regulator improves the grain yield of high-density maize crops by reducing stalk lodging and promoting a compact plant type. Field Crop Res. 260 (1), 107982. doi: 10.1016/j.fcr.2020.107982

[B12] Hu C. H. Zheng Y. Tong C. L. Zhang D. J. (2022). Effects of exogenous melatonin on plant growth, root hormones and photosynthetic characteristics of trifoliate orange subjected to salt stress. Plant Growth Regul. 97, 551–558. doi: 10.1007/s10725-022-00814-z

[B13] Jiang H. Q. Xing X. H. Zhou Q. Jiang H. D. (2015). Effects of exogenous α-naphthaleneacetic acid on the antioxidation system in soybean leaves subjected to long-term drought stress during flowering. Chin. J. Appl. Ecol. 26 (6), 1718–1726. 26572024

[B14] Kazuhisa K. Yoshimichi O. Koki K. Kanayama Y. (2003). Nitrate-independent expression of plant nitrate reductase in *Lotus japonicus* root nodules. J. Exp. Bot. 54 (388), 1685–1690. doi: 10.1093/jxb/erg189 12773524

[B15] Khan T. Ullah S. Shuaib M. Alsamadany H. Alzahrani Y. Alharbi N.. (2018). Effect of nephthyl acetic acid foliar spray on amelioration of salt stress tolerance in maize *Zea mays* l. Appl. Ecol. Env. Res. 17 (2), 1817–1834. doi: 10.15666/AEER/1702_18171834

[B16] Kim S. E. Schlicke H. Vanree K. Karvonen K. Subramaniam A. Richter A.. (2013). Arabidopsis chlorophyll biosynthesis: an essential balance between the methy-lerythritol phosphate and tetrapyrrole pathways. Plant Cell. 25 (12), 4984–4993. doi: 10.1105/tpc.113.119172 24363312 PMC3904000

[B17] Kumar S. Ayachit G. Sahoo L. (2020). Screening of mungbean for drought tolerance and transcriptome profiling between drought-tolerant and susceptible genotype in response to drought stress. Plant Physiol. Biochem. 157, 229–238. doi: 10.1016/j.plaphy.2020.10.021 33129069

[B18] Liang Y. F. Wang K. C. Xue Q. Sui L. Ye J. Chen X. Z.. (2017). Effects of exogenous growth substances on physiological traits of cold tolerance in *Citrus aurantium* seedlings. Scientia Silvae Sinicae. 53 (3), 68–75.

[B19] Li J. Guan Y. L. Yuan L. Y. Hou J. F. Wang C. G. Liu F. F.. (2019). Effects of exogenous IAA in regulating photosynthetic capacity, carbohydrate metabolism and yield of zizania latifolia. Sci. Hortic. 253, 276–285. doi: 10.1016/j.scienta.2019.04.058

[B20] Li P. Li B. Y. Seneweera S. Zong Y. Z. Li F. Y. Han Y.. (2019). Photosynthesis and yield response to elevated CO_2_, C_4_ plant foxtail millet behaves similarly to C_3_ species. Plant Science. 285, 239–247. doi: 10.1016/j.plantsci.2019.05.006 31203889

[B21] Liu T. Yang X. G. Gao J. Q.. (2020). Radiation use efficiency of different grain crops in northeast China. Trans. Chin. Soc. Agric. Eng. (Transactions CSAE). 36 (24), 186–193.

[B22] Liu H. J. Zhang C. X. Wang J. M. Zhou C. Feng H. Mahajan M. D.. (2017). Influence and interaction of iron and cadmium on photosynthesis and antioxidative enzymes in two rice cultivars. Chemosphere 171, 240–247. doi: 10.1016/j.chemosphere.2016.12.081 28024209

[B23] Luo K. Xie C. Wang J.. (2021). Uniconazole, 6-benzyladenine, and diethyl aminoethyl hexanoate increase the yield of soybean by impro-ving the photosynthetic efficiency and increasing grain filling in maize-soybean relay strip intercropping system. J. Plant Growth Regul. 40 (5), 1869–1880. doi: 10.1007/s00344-020-10236-8

[B24] Makino A. (2011). Photosynthesis, grain yield, and nitrogen utilization in rice and wheat. Plant Physiol. 155, 125–129. doi: 10.1104/pp.110.165076 20959423 PMC3014227

[B25] Martins J. P. R. Rodrigues L. C. A. Santos E. R.. (2018). Anatomy and photosystem II activity of *in vitro* grown *Aechmea blanchetiana* as affected by 1-naphthaleneacetic acid. Biol. Plantarum. 62 (2), 211–221. doi: 10.1007/s10535-018-0781-8

[B26] Matthew T. H. Duke P. Todd C. M. Thompson A. L. (2020). Chlorophyll fluorescence imaging captures photochemical efficiency of grain sorghum (*Sorghum bicolor*) in a field setting. Plant Methods 16 (1), 109. doi: 10.1186/s13007-020-00650-0 32793296 PMC7419188

[B27] Ma G. Zhang L. Kudaka R. Inaba H.. (2021). Auxin induced carotenoid accumulation in GA and PDJ-treated citrus fruit after harvest. Postharvest Biol. Technol. 181, 111676. doi: 10.1016/j.postharvbio.2021.111676

[B28] Prasad F. Prakash V. Jilani A. Prasad S. Thomas M. R. Arnold R.. (2014). Effect of naphthalene acetic acid (NAA) as growth regulator along with chelated zinc and iron on the availability of manganese, zinc, copper and iron in mentha piperita Linn cultivar kukrail. Int. J. Environ. 3 (2), 238–245. doi: 10.3126/ije.v3i2.10636

[B29] Qamar R. Khan S. Safdar M. E. Atique U. R. Rehman A. Javeed H. M.R.. (2022). Seed priming with growth regulators modulates production, physiology and antioxidant defense of Indian squash (*Praecitrullus fistulosus*) under semi-arid conditions. PloS One 17 (4), e0265694. doi: 10.1371/journal.pone.0265694 35421113 PMC9009649

[B30] Qu Y. Y. Yu J. H. Tao X. L. Chang T. (2006). Effect of seed soaked with S_3307_ and IBA +NAA on cold resistance of sweet pepper seedlings. J. Gansu Agric. University. 41 (004), 52–55.

[B31] Rohach V. V. (2017). Influence of growth stimulants on photosynthetic apparatus, morphogenesis and production process of egg-plant (*Solanum melongena)* . Biosyst. Diversity. 25 (4), 297–303. doi: 10.15421/011745

[B32] Salehifar M. Rabiei B. Afshar M. M. Asghari J. (2017). Physiological and fluorescence reaction of four rice genotypes to exogenous application of IAA and kinetin under drought stress. Notulae Scientia Biologicae. 9 (3), 378–385. doi: 10.15835/nsb9310091

[B33] Shi L. Yuan T. T. Xu Z. Q. Xie Y. F. (2021). Effects of naphthylacetic acid on photosynthetic characteristics of pseudostellaria heterophylla. Acta agriculturae universitatis Jiangxiensis. 43 (2), 253–260.

[B34] Tang S. Shahriari M. Xiang J. Pasternak T. Igolkina A. Aminizade S.. (2022). The role of AUX1 during lateral root development in the domestication of the model C4 grass *Setaria italica* . J. Exp. Botany. 73, 2021–2034. doi: 10.1093/jxb/erab556 34940828

[B35] Wang Y. Y. (2019). “Studies on autotoxicity and mitigation measures of foxtail millet continuous cropping obstacles,” in Shanxi agricultural university master degree dissertation. doi: 10.11841/j.issn.1007-4333.2019.06.05

[B36] Wang S. L. Guo T. C. Wang C. Y. Cha F. N. Song X. (2005). Soluble sugar contents in leaf and grain in two gluten wheats and its relationship with grain starch accumulation. J. @ Henan Agric. Sci. 4, 12–15.

[B37] Wen G. Q. Nong M. L. Liu Y. X.. (2016). Effects of plant growth regulators (combination) on chlorophyll content and amylolysis of yam. Southwest China J. Agric. Sci. 29 (6), 1281–1284. doi: 10.16213/j.cnki.scjas.2016.06.008

[B38] Xu C. Gao Y. Tian B. Ren J. Meng Q. Wang P.. (2017). Effects of EDAH, a novel plant growth regulator, on mechanical strength, stalk vascular bundles and grain yield of summer maize at high densities. Field Crop Res. 200 (10), 71–79. doi: 10.1016/j.fcr.2016.10.011

[B39] Yuan Y. J. Mei L. H. Wu M. B. Wei W. Shan W. Gong Z.. (2018). SlARF10, an auxin response factor, is involved in chlorophyll and sugar accumulation during tomato fruit development. J. Exp. Bot. 69 (22), 5507–5518. doi: 10.1093/jxb/ery328 30219898 PMC6255703

[B40] Zhao S. W. Wang J. Y. (2012). Comprehensive prevention and treatment of downy mildew of foxtail millet. Agric. Technol. Equipment. 10, 44–45.

[B41] Zheng J. Yang C. Zheng X. Yan S. Qu F. Zhao J.. (2021). Lipidomic, transcriptomic, and BSA-660K single nucleotide polymorphisms profiling reveal characteristics of the cuticular wax in wheat. Front. Plant Sci. 12, 794878. doi: 10.3389/fpls.2021.794878 34899814 PMC8652291

